# Factorial and Economic Evaluation of an Aqueous Two-Phase Partitioning Pilot Plant for Invertase Recovery From Spent Brewery Yeast

**DOI:** 10.3389/fchem.2018.00454

**Published:** 2018-10-02

**Authors:** Patricia Vázquez-Villegas, Edith Espitia-Saloma, Mario A. Torres-Acosta, Federico Ruiz-Ruiz, Marco Rito-Palomares, Oscar Aguilar

**Affiliations:** ^1^Escuela de Ingenieria y Ciencias, Tecnologico de Monterrey, Monterrey, Mexico; ^2^Escuela de Medicina y Ciencias de la Salud, Tecnologico de Monterrey, Monterrey, Mexico

**Keywords:** aqueous two-phase system, biosolve, invertase, operational parameters, pilot plant, spent yeast

## Abstract

Aqueous two-phase systems (ATPS) have been reported as an attractive biocompatible extraction system for recovery and purification of biological products. In this work, the implementation, characterization, and optimization (operational and economic) of invertase extraction from spent brewery yeast in a semi-automatized pilot plant using ATPS is reported. Gentian violet was used as tracer for the selection of phase composition through phase entrainment minimization. Yeast suspension was chosen as a complex cell matrix model for the recovery of the industrial relevant enzyme invertase. Flow rates of phases did not have an effect, given that a bottom continuous phase is given, while load of sample and number of agitators improved the recovery of the enzyme. The best combination of factors reached a recovery of 129.35 ± 2.76% and a purification factor of 4.98 ± 1.10 in the bottom phase of a PEG-Phosphate system, also resulting in the removal of inhibitor molecules increasing invertase activity as reported by several other authors. Then, an economic analysis was performed to study the production cost of invertase analyzing only the significant parameters for production. Results indicate that the parameters being analyzed only affect the production cost per enzymatic unit, while variations in the cost per batch are not significant. Moreover, only the sample load is significant, which, combined with operational optimization results, gives the same optimal result for operation, maximizing recovery yield (15% of sample load and 1 static mixer). Overall res ults of these case studies show continuous pilot-scale ATPS as a viable and reproducible extraction/purification system for high added-value biological compounds.

## Introduction

With the improvement in recombinant technologies, great progress has been taking place in the upstream processes of bioproducts manufacturing. However, with the increasing of cell culture titers the downstream processing capacity arises as a bioprocessing bottleneck (Lowe, 2001; Rosa et al., 2010; Langer, 2011; Straathof, 2011; Dizon-Maspat et al., 2012; Sabalza et al., 2014). The currently available purification technology typically include unit operations with limited operational windows or scaling up drawbacks, besides the use of high cost equipment (Xu et al., 2003; Aguilar et al., 2010; Rosa et al., 2011; Goja et al., 2013). This has promoted the development and implementation of alternative, easily scalable extraction procedures and purification systems that allow higher throughputs.

During the last decades, several research groups have consistently reported the advantages that aqueous two-phase systems (ATPS) possess as an extraction/purification technique: they show biological compatibility, high load capacity, scale-up easiness and continuous operation, among others (Raghavarao et al., 2003; Benavides and Rito-Palomares, 2008; Rosa et al., 2010; Asenjo and Andrews, 2012)]. There are some works on semi-continuous/pilot plant ATPS (Minuth et al., 1997; Huenupi et al., 1999; Kepka et al., 2003; Igarashi et al., 2004; Rosa et al., 2009; Sutherland et al., 2011), however, most of the practical attempts to apply ATPS have been carried out in batch mode or at bench scale. This makes continuous operation at pilot scale an open research area for the implementation of ATPS processes (Rosa et al., 2010; Benavides et al., 2011; Espitia-Saloma et al., 2014). Continous operation is acknowledged for its positive impact in processing time, costs and yields, having the potential to make bioproducts economically competitive at larger scales (Igarashi et al., 2004; Benavides et al., 2011). Conventional equipment used by the chemical industry for organic-aqueous extraction, such as column contactors, has been mostly employed for large-scale and continuous ATPS applications (Cuhna and Aires-Barros, 2002). However, the study of alternative equipment configurations such as mixer settlers and the development of novel separators for different ATPS variants, bioproducts and physicochemical characteristics could increase the interest toward the industrial implementation of continuous ATPS-based processes (Cuhna and Aires-Barros, 2002; Benavides and Rito-Palomares, 2008; Torres-Acosta et al., 2016). Recently, Vázquez-Villegas et al. (2011), proposed a novel mixer-settler device for continuous ATPS operation at bench scale. It is based on a tubular reactor approach with a large and adjustable length/diameter ratio to control settling time and separation of phases (Vázquez-Villegas et al., 2011).

Pilot plant studies, are of noteworthy value in the development of new processes, since they allow the study, in an efficient and relatively economic way, of different technical aspects (operation conditions, design parameters, construction materials, corrosion, and operational procedures) essential for any industrial process. The objective of this work is to present a continuous ATPS-based process at pilot plant level, and evaluate its operational and economic performance for the extraction of invertase from spent brewery yeast.

## Materials and methods

### Chemicals and biological material

Polyethylene glycol with molecular weight of 1,000 Da (PEG1000) was purchased from Avizor Química (Monterrey, Mexico). Gentian violet (GV) and bovine serum albumin (BSA) were obtained from Sigma Aldrich (St. Louis, MO, USA). Monobasic and dibasic potassium phosphate and other analytical grade reagents were purchased from Desarrollo de Especialidades Químicas (DEQ, Monterrey, Mexico). Spent brewery yeast was kindly donated by Cervecería Cuauhtémoc-Moctezuma, S.A. de C.V (Nuevo León, México).

#### Spent brewer's yeast preparation

Spent brewer's yeast, directly obtained from brewery, was centrifuged at 4,300 rpm at 4°C for 10 min (Thermo Scientific IEC CL40R, WaltHam, USA). The supernatant was discarded. The biomass was resuspended (40% w/v) in 50 mM phosphate buffer at pH 7. Cell disruption was accomplished in a bead mill with 0.5 mm glass beads (Dyno Mill-Multi Lab, Muttenz, Switzerland). The grinding chamber was initially filled with a 50% v/v bead load and the biomass was fed with a peristaltic pump (Watson–Marlow 323 S/D) at 5 mL/min and recirculated for 15 min. Temperature was kept constant at 4°C. The disrupted cell suspension was directly loaded into the ATPS.

### Preparation of ATPS

Two basic ATPS compositions were studied for their influence in performance of the pilot plant based on previous experiences, System 1: 17% w/w PEG 1000 and 15.2% w/w potassium phosphates and System 2: 14% w/w PEG 1000 and 18% w/w potassium phosphates. In all the cases the potassium phosphates mixture was composed of a 1.82:1 dibasic and monobasic potassium phosphates ratio to obtain a pH of 7. For the stock solutions, potassium phosphate and PEG 1000 were weighed separately and mixed with the appropriate amount of distilled water until complete dissolution.

### Batch ATPS

In order to compare the performance of the continuous system, ATPS batch systems were prepared with the same volume and composition as the total processed volume in the pilot plant continuous system (9 L of bottom phase and 3 L of top phase). The sample was pre-dissolved in the total initial bottom phase at the same concentration as the one employed in the continuous system. Afterwards, the top phase was added and the system was stirred for 10 min. At the end of the mixing stage samples from the top and bottom phases of the batch system were taken manually from the middle of each phase bulk every minute.

### Pilot plant configuration and operating procedure

**Figure 1:**
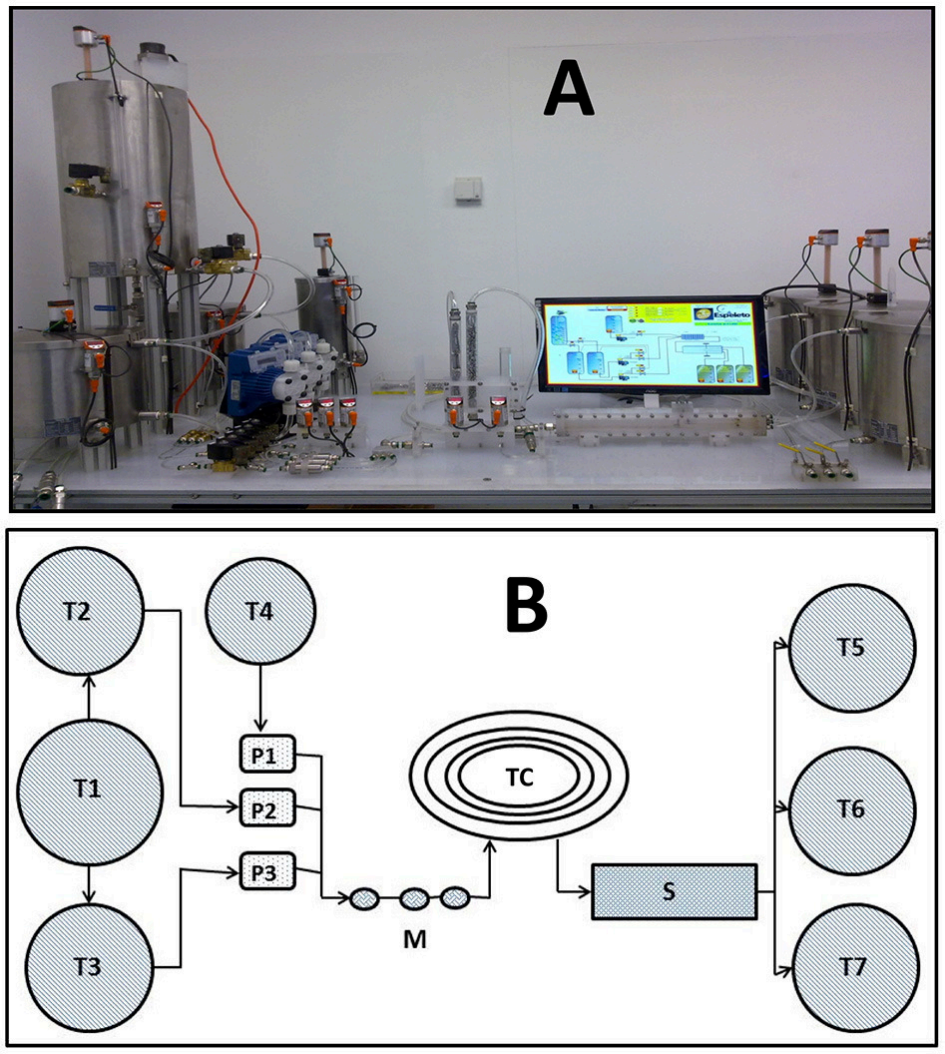
Front view of the pilot plant configuration **(A)**. Top view diagram **(B)** showing the main elements: Feeding tanks (**T1**, **T2**, **T3**, **T4**), Pumps (**P1**, **P2**, **P3**), Static mixers (**M**), Tubular Coalescer (**TC**), Gravity Separator (**S**), Recollecting tanks (**T5**, **T6**, **T7**).

The pilot plant prototype was manufactured in stainless steel by Patmon Automatización (Nuevo León, México). As shown in Figures 1A,B it consists of 5 main stages: ATPS formulation and storage of feeding stocks, a series of in-line static mixers (adaptable from 1 to 4 static mixers), an adaptable length polyurethane tubular coalescer (6.5 m long), a liquid-liquid gravity separator with three outlets for recollecting top, bottom phase and interface and three final phase storage tanks. Phases and sample are fed with three digital solenoid dosing metering pumps with maximum capacity of 1.5 L/min (Tekna Evo, Seko GmbH, Kastel, Germany). The static mixers are conformed of acrylic cylindrical columns with a stainless steel screw and nut packing. The gravity separator consists of a rectangular container (round shaped inner corners) with one inlet and three vertical aligned outlets. Additional information about the pilot plant dimensions can be found in Table 1. Pilot plant operation was semi-automatized using a programmable logic controller (PLC) (IFM Efector, Nuevo León, México) allowing independent control of the flows.

**Table 1:** Dimensions of the scaled-up prototype components.

**Pilot plant component**	**Dimension**
Feeding tanks	TP tank (T2) volume: 16 L BP tank (T3) volume: 16 L Sample tank (T4) volume: 5.5 L
Static mixers	Length: 23.5 cm Internal diameter: 2 cm Void volume: 43.8 cm^3^
Phase coalescer	Internal diameter: 1 cm Length: adaptable (3-6 m)
Separator	Inlet diameter: 1.27 cm Outlets diameter: 0.64 cm Length: 43 cm Width: 7.7 cm Height: 7.7 cm Internal volume: 800 mL
Final recollecting tank	TP tank (T5) volume: 23 L IP tank (T6) volume: 23 L BP tank (T7) volume: 23 L

Pilot plant runs were carried out at room temperature (25°C). The corresponding ATPS were mixed and equilibrated in tank T1. Afterwards top and bottom phases were separated through solenoid valves into T2 and T3 respectively. Starting in T4, the disrupted cell suspension was fed independently. Knowing its density as well of that of both phases, the feeding flow was setted up. Samples were taken every minute from the outlets to the final recollecting tanks during 30 min runs.

### Evaluation of operational performance

Phase entrainment profile (defined as the amount of carry-over of one phase into the other) was first studied with the two different system compositions, using a dye (gentian violet) as tracer (Giraldo-Zuniga et al., 2006). Entrainment behavior was compared with a batch system to choose the appropriate system composition. Dye concentration in top phase was also measured (580 nm, Bio-Tek Instruments, VT, U.S.A.). After the selection of the system that generated lower entrainment in the bottom phase, a duplicate full 2^4^ factorial design augmented with 6 center points was carried out (Table 2). This experimental design was implemented in order to study the effect of phases flow rates, number of static mixers and load of sample over invertase purification factor (PF_BP_) and activity recovery (R_BP_) in bottom phase as well as entrainment in that phase (E_*BP*_), calculated as:

(1) $$\begin{array}{rcl} PF_{BP} & = & \frac{{\left(\frac{EA}{Total\;protein}\right)}_{BP}}{{\left(\frac{EA}{Total\;protein}\right)}_{0}} \end{array}$$

(2) $$\begin{array}{rcl} R_{BP} & = & \frac{EA_{BP}}{EA_{0}} \end{array}$$

(3) $$\begin{array}{rcl} E_{BP} & = & \frac{V_{TP}}{V_{TP}+V_{BP}} \end{array}$$

**Table 2:** Variables and levels of the central composite design (CCD) carried out for enzyme recovery from spent brewer's yeast using the pilot plant prototype.

**Studied variables**		**Levels**	
		**-1**	**1**
x_1_	Top phase feed flow (mL/min)	100	300
x_2_	Bottom phase feed flow (mL/min)	200	400
x_3_	Sample load percentage	5	15
x_4_	Number of static mixers	1	3

Where EA_0_ represents the enzymatic activity on the disrupted yeast supernatant, while V_BP_ represents the bottom phase volume and V_TP_ represents the top phase volume measured in the final recollecting tanks of the pilot plant. All experiments and replicates were modeled and analyzed with JMP 14.1.0 data software (SAS Institute, Cary, NC, USA) and response surface using Minitab 18 software (Minitab Inc.).

### Evaluation of economic performance

To perform an economic analysis, the commercial software platform Biosolve Process (Biopharm Services, Chesham, Buckinghamshire, UK) was used. For this, the methodology presented before was followed (Torres-Acosta et al., 2016). Briefly, a model was created with which a determinisitic analysis was done to obtain the production cost per batch (CoG/batch) and cost per enzymatic unit (CoG/EU). Then production parameters were varied only in the selected ATPS from the previous section to analyze from an economic perspective the significant results from the factorial design experiments.

To create a model for the production of invertase, the process was simplified into a single unit operation which processed the total amount of sample and materiales required for the operation, then it was calculated the production cost per batch (CoG/batch) and per enzymatic unit (CoG/EU). To set up the model, the data presented in Table 3 was used. Briefly, to fully construct a model, four main datasets should be completed: (1) capital, (2) materials, (3) consumables, and (4) labor.

**Table 3:** Dataset used for model construction in Biosolve Process.

	**Cost component**	**Cost (US $)**	**Supplier**
Capital *(Equipment)*	Main equipment	$ 35,240.90	Patmon Automatizacion
	Static mixer	$ 836.27	USA Blue Book
Consumables	None Required	$ –	None
Labor	Annual Salary (PhD Student in Mexico)	$ 8,520.00	Conacyt (Mexican Science Council)
Materials *(Prices used were obtained for large-laboratory scale to overestimate worldwide distribution)*	Polyethylene Glycol 1,000 (1 kg)	$ 56.20	Sigma-Aldrich
	Potassium phosphate monobasic (20 kg)	$ 607.70	Sigma-Aldrich
	Potassium phosphate dibasic (10 kg)	$ 1,020.00	Sigma-Aldrich

Capital was calculated only taking into account equipment cost, as this process is performed in an already equipped space and only the acquisition of the main device and its static mixers for the performance of continuous ATPS were required. To obtain the capital charge per batch or per enzymatic unit, the cost of the equipment required was treated as a loan with 12% interest rate with a 10 years duration. Then it was divided by the year duration and by batches calculated per year (2.5 h per ATPS run−30 min runs plus setup and cleaning time).

Materials for this model only include those involved in the construction of the systems employed here (Polyethylene glycol 1,000 Da and monobasic and dibasic potassium phosphate). This particular process has the advantage of not requiring consumables, contrasted to other unit operations, like chromatography or different filtration types. Lastly, labor was calculated using the salary of a PhD student, as production was done like this in the pilot plant. Additionally, Biosolve is able to integrate a fifth area called “Others,” in which maintance, waste disposal and utilities costs are automatically calculated. For this model it was calculated to be approximately 4.5% of the CoG/EU.

### Analytical techniques

Invertase enzymatic activity was determined by the 3,5-dinitrosalycilic acid method (DNS, 98%, Sigma Aldrich) (Miller, 1959). Absorbance was read at 570 nm in a microplate spectrophotometer (Biotek, Vermont, USA). One unit of enzymatic activity (U) was defined as the amount of enzyme necessary to produce 1 μmol of glucose per minute in the assay reaction conditions. Total protein was calculated using the Bradford method and a calibration curve using BSA as protein standard (Bradford, 1976).

## Results and discussion

The knowledge of the system hydrodynamics is essential in extraction processes. Usually, the performance of liquid-liquid extraction systems can be affected for unwanted side effects related to hydrodynamic parameters (Asadollahzadeh et al., 2017). As an example, while low phase entrainment results in higher yields, some works, have demonstrated that mass transfer is improved in simplified equipments where the operating range is increased (Glyck et al., 2015). In recent years, the hydrodynamic parameters of different kinds of extraction columns have been investigated by several researchers but not for ATPS systems in pilot plant scale. In the present work, the design of a pilot plant ATPS continuous process has been simplified and optimized in terms of separation efficiency, load capacity, and economic suitability for downstream processing of spent brewery yeast as a model system.

**Figure 2:**
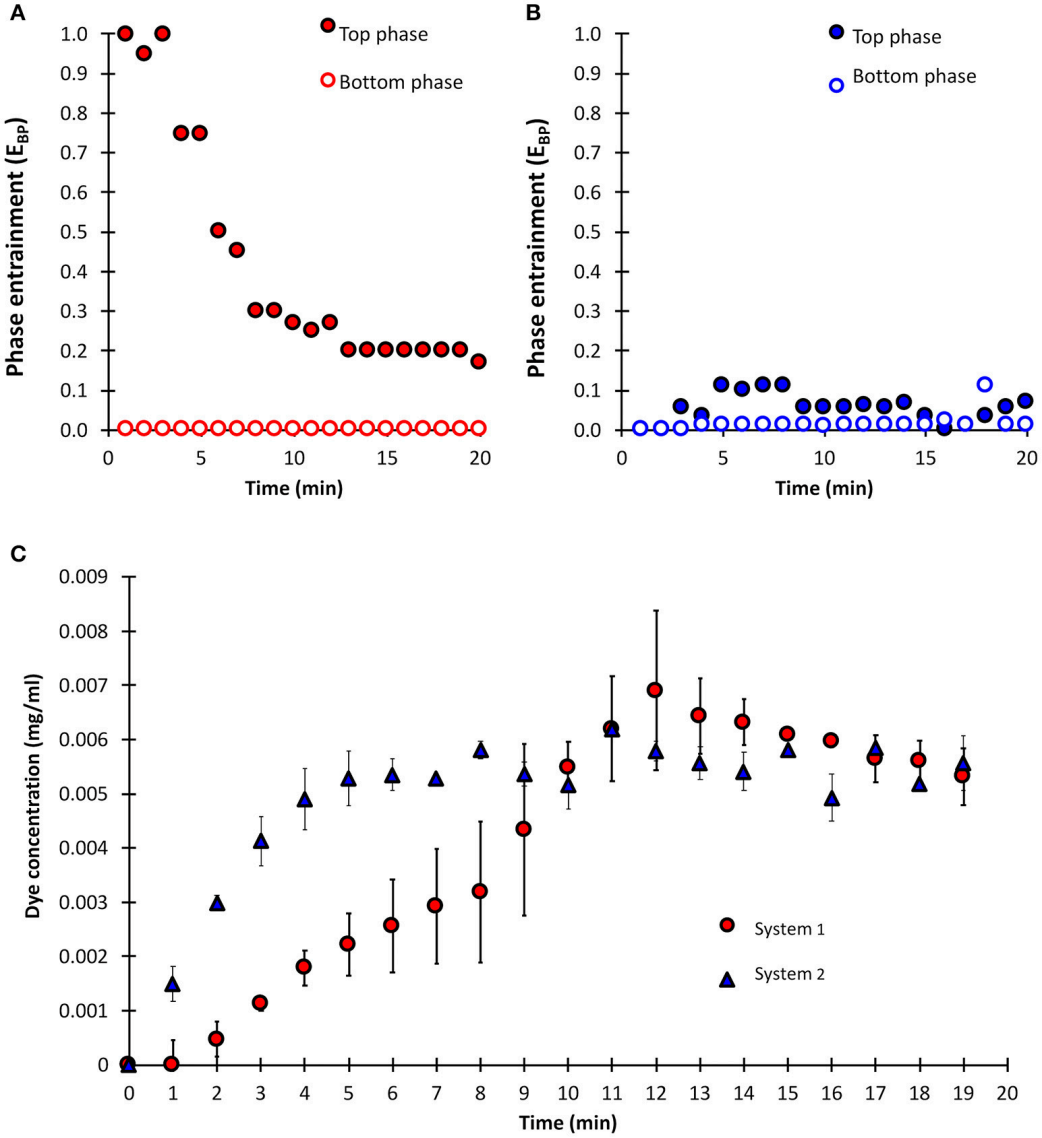
Phase entrainment (E_*BP*_) comparison between System 1 **(A)** and System 2 **(B)**. Concentration of gentian violet used as tracer in top phase of Systems 1 and 2 **(C)**.

Considering two ATPS compositions previously explored for the recovery of enzymes (Vázquez-Villegas et al., 2011, 2015), phase entrainments were compared. Top phase fed (TP) at 100 mL/min and bottom phase fed (BP) at 300 mL/min were employed. The stabilization time was defined as the time in which entrainment (E_*BP*_) of bottom phase (or top phase) in the opposite phase does not change significantly for a period of 5 min. Entrainment was defined constant when the change is no more than 10% of the previous value. For System 1 the stabilization time was ca. 20 min, while for System 2 the time for complete separation of phases was almost immediate (Figures 2A,B). In this case, a larger difference between the composition of the two phases (typically expressed as the tie-line length value, TLL) benefits the equilibrium and separation of the ATPS yielding shorter processing times. This increment in the demixing rate as consequence of a higher TLL agrees with previous observations (Salamanca et al., 1998; Aguilar and Rito-Palomares, 2008; Narayan et al., 2011). Just for comparison, the concentration of GV in top phase was monitored. While the dye concentration was the same at the end of the sampling, a faster equilibrium was observed also in the System 2 (Figure 2C). So, this system was selected for the next experiments. The influence of physicochemical parameters on the separation efficiency when using mixer settler devices has been previously studied (Salamanca et al., 1998; Vázquez-Villegas et al., 2011). In these cases, as in this pilot plant, the operating volume ratio of the phases (defined as the ratio of top phase between bottom phase feeding flows) defines which of the phases acts as the continues or the dispersed phase, where the viscosities play a determinant factor for phase separation. A continuous salt phase, being much less viscous than the polymer phase, favors shorter separation times by lowering the friction between drops and the phase (Asenjo and Andrews, 2012).

Spent brewer's yeast was used as model of complex cellular matrix to challenge the recovery of invertase with the continuous pilot-scale device. Anticipating an altered hydrodynamic behavior of the effluents, due to the complex matrix addition, a full 2^4^ factorial experimental design with six central points was performed (Table 2). The experimental design allowed the analysis of the partitioning behavior patterns due to changes in the operation parameters. The ideal scenario for any enzyme will be having the optimal conditions for maximizing purity and recovery. In the case of invertase, when the results of different factor combinations were analyzed, an average of 110.98 ± 28.44 % for recovery and 4.47 ± 2.46 for purification fold in the bottom phase were obtained as a total average from the full factorial runs. The high variability observed is explained by the difference in response obtained at different factor levels. Enzyme activity recoveries above 100% have been extensively documented for invertase and other enzymes when recovered using ATPS (Cavalcanti et al., 2006; Babu et al., 2008; Porto et al., 2008; Madhusudhan and Raghavarao, 2011; Nandini and Rastogi, 2011; Rawdkuen et al., 2011; Karkas and Onal, 2012; Priyanka et al., 2012; Duque-Jaramillo et al., 2013; Ketnawa et al., 2014, 2017) and this phenomenon is attributed to the depletion of inhibiting proteins and common enzyme inhibitors by differential partitioning. Common identified inhibitors include Cu, Co and Ni salts which become insoluble in the presence of the high phosphate concentrations in ATPS. Enzyme activation has also been observed in ATPS due to an increase in the enzyme flexibility and the structural modification of the active sites in the presence of polymer chains of PEG (Babu et al., 2008; Porto et al., 2008; Madhusudhan and Raghavarao, 2011; Nandini and Rastogi, 2011; Karkas and Onal, 2012). Cavalcanti et al. (2006) in extraction studies of phospholipase C, used a similar PEG/phosphate ATPS and reported yields up to 230% attributed to removal of inhibiting phenoic compounds (Duque-Jaramillo et al., 2013).

**Figure 3:**
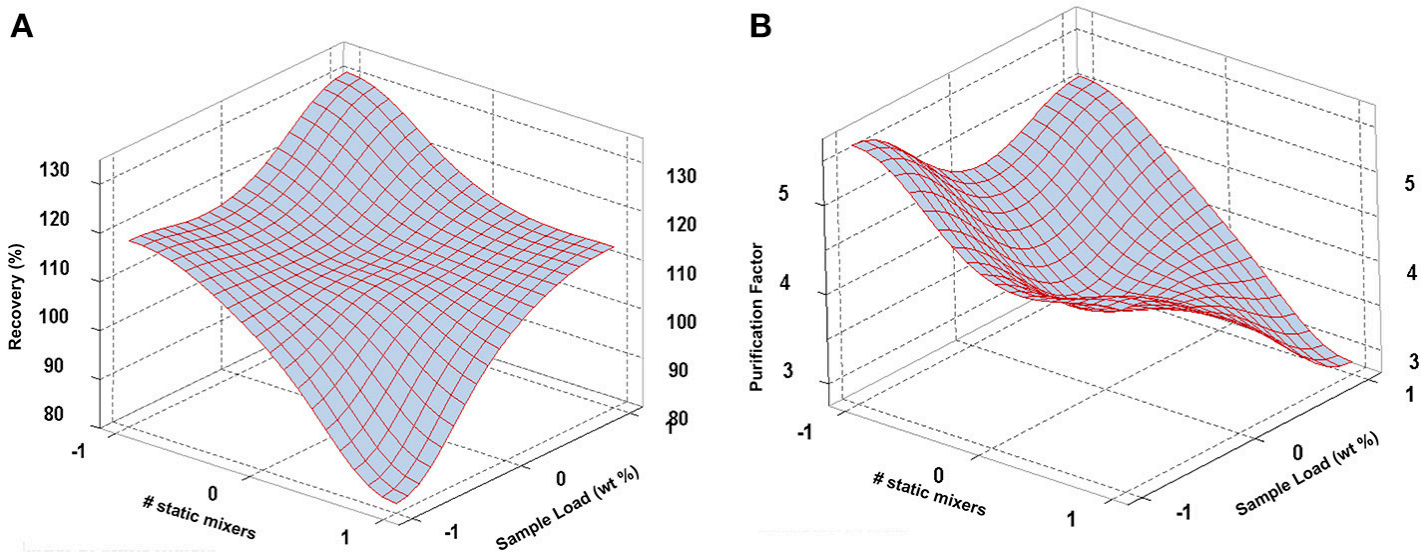
Response surface plots for recovery yield **(A)** and purification factor **(B)** of invertase from spent brewer's yeast considering the effect of sample load (X3) and number of static mixers (X4).

The best combination of factors (1 mixer and 15% sample load) attained a recovery of 129.35 ± 2.76% and a purification factor of 4.98 ± 1.10 in the bottom phase of a PEG-phosphate system; however, only the sample load and the number of static mixers were significant, thus a surface response analysis was performed. As observed in Figure 3, higher sample load yielded major recoveries and slightly minor purity, as it would naturally occur, while for the number of mixers, the use of one static mixer yielded higher values for both recovery and purification factor. This would mean that the higher the mixing rate, for this particular system, the lower the efficiency of the pilot plant in terms of recovery and purification. This could be better explained when considering the amount of other contaminants also partitioning or dispersing into the bottom phase, promoted by higher mixing rates. Also, higher mixing promotes lower coalescence rates that could affect separation efficiency resulting in the lower purification observed in Figure 3B (Asenjo and Andrews, 2012). The high statistical variability of purification factor could also be the result of poor separation efficiency. For invertase, other studies have shown a tendency to partition toward the bottom phase in PEG/salt systems (Madhusudhan and Raghavarao, 2011; Karkas and Onal, 2012; Vázquez-Villegas et al., 2015), as it was observed in the present work. Taking this into account, the economic analysis was carried out.

The economic analysis performed obtained interesting results that further help to elucidate which parameters are relevant for the continuous operation of an ATPS pilot plant. With the results presented here (Table 4), linear regressions were calculated to summarize the effect of each variable and to determine which are economically significant. Results show that the only significant parameter is the sample load, while number of static mixers is not (Table 5). This can be explained by the fact that increasing the load of sample directly affects the amount of invertase being recovered, moreover, an increase in sample load gave as a result an increased recovery yield. On the other hand, modifying the amount of static mixers increased the capital charge in the cost but given the elevated cost of the main equipment, variations in the cost due to modifying the number of mixers are diluted.

**Table 4:** Results for the economic analysis. Breakdown and totals for cost of Goods per Enzymatic Unit.

**CoG/EU (US$ x 10**^**∧**^**-3)**								
**Sample load**	**Static mixers**	**Recovery yield**	**Capital**	**Materials**	**Consumables**	**Labor**	**Other**	**Total**
5%	1	115%	$0.34	$15.86	$ –	$4.91	$1.01	$22.12
5%	3	80%	$0.51	$22.79	$ –	$7.06	$1.46	$31.82
15%	1	142%	$0.09	$4.28	$ –	$1.33	$0.27	$5.97
15%	3	108%	$0.13	$5.63	$ –	$1.74	$0.36	$7.86

**Table 5:** Linear regression results.

Parameters*	**Coefficient**	***p*-value**	**Significant?^a^**
*Intercept (*β_0_*)*	29.71	0.0292	Yes
*Sample Load (*β_1_*)*	−1.71	0.0339	Yes
*Static Mixers (*β_2_*)*	1.39	0.2001	No

* *Equations of linear regression have the form: CoG/EU [US$ x10-3] = β0 + β1 x Sample Load [%] + β2 x Static Mixers [Amount of mixers]*.

a *Using a significance level α = 0.05*.

From an economic perspective, it is relevant to maximize the sample load in an ATPS. It has been discussed before (Aguilar and Rito-Palomares, 2008; Torres-Acosta et al., 2015, 2016) that due to low sample input into an ATPS, costs cannot be further decreased. Sample load increases cannot be used lightly in bioprocess modeling as the physics underlying protein separation are too complex to model and it is not certain the effect that modifying the amount of sample can have on the recovery yield and purification factor. However, having a proper analysis of sample input in this work, its impact in the economic analysis can be determined and quantified.

Further analysis is required to contrast continuous and batch operation modes. Still, results for recovery and economics together are promising, moreover if operational parameters can be further optimized production costs will drop. Having a continues ATPS, together with a phase forming chemical removal operation synchronized can significantly reduce production times and costs.

Novel strategies for the high throughput design of ATPS-based processes (microfluidic modeling) as well as different modes of operations (countercurrent modes) could also be considered in the process to optimize performance of continuous processes (Espitia-Saloma et al., 2016; Vázquez-Villegas et al., 2016). This work highlights the importance of the influence of the system properties on the hydrodynamics of the bioseparation process. In some case studies, TLL has little or no influence on the partition behavior of the desired product (Aguilar and Rito-Palomares, 2008), allowing the selection of the best system in terms of the hydrodynamic behavior in the equipment. It should be emphasized that even in a batch process the phase separation stage would imply a phase entrapment, meaning a carry-over of one phase into the other, and time (or a centrifuge) needed for the physical separation. In this continuous mode, the physical separation of the phases is already accounted as part of the stabilization time, the time in which the evaluated parameter (E_*i*_) begins to be constant.

## Conclusions

The recovery of products of interest from fermentation broths and biological feedstock is one of the major bottlenecks in the bioprocessing industries. This article introduces a first approach for continuous pilot plant ATPS operation and process establishment. A robust, large-scale, automated process able to be managed by simple development strategies such as factorial and central composite designs was established in this work.

A semi-automatized mixer settler pilot plant with a maximum capacity of 1.5 L/min was characterized. The use of single molecule samples allowed to demonstrate some of the recognized advantages of continuous operation over batch mode. Short stabilization times inside the coalescer avoid the use of centrifugation steps, as typically suggested in batch processes, also contributing to process integration, as evidenced by processing a complex sample such as non-clarified spent brewer's yeast. Different combination of hydrodynamic operation parameters allowed the high yield recovery and the highest purification fold of invertase from a complex cell lysate. Adjustments in phases flows did not altered the extraction efficiency of the pilot plant. This works serves to boost the application of ATPS in different operation modes by combining a techno-economic approach to evaluate and optimize production parameters that can facilitate ATPS generic and commercial adoption.

## Author contributions

PV-V, EE-S, and FR-R performed experiments with pilot plant equipment, analytical assays, and wrote the paper. MT-A and FR-R performed economical analysis of the results. MR-P and OA designed the research, analyzed data and revised economical analysis performed. All authors read and approved the final manuscript.

### Conflict of interest statement

The authors declare that the research was conducted in the absence of any commercial or financial relationships that could be construed as a potential conflict of interest.
